# Interpretation der Photoplethysmographie: Schritt für Schritt

**DOI:** 10.1007/s00399-021-00795-y

**Published:** 2021-07-24

**Authors:** Konstanze Betz, Rachel van der Velden, Monika Gawalko, Astrid Hermans, Nikki Pluymaekers, Henrike A. K. Hillmann, Jeroen Hendriks, David Duncker, Dominik Linz

**Affiliations:** 1 Department of Cardiology, Maastricht University Medical Centre and Cardiovascular Research Institute Maastricht, Maastricht UMC+, 6202 AZ Maastricht, Niederlande; 2 Hannover Heart Rhythm Center, Department of Cardiology and Angiology, Hannover Medical School, Hannover, Deutschland; 3 Caring Futures Institute, College of Nursing and Health Sciences, Flinders University, Adelaide, Australien; 4 Department of Cardiology, Radboud University Medical Centre, Nijmegen, Niederlande; 5 Centre for Heart Rhythm Disorders, University of Adelaide and Royal Adelaide Hospital, Adelaide, Australien; 6 Department of Biomedical Sciences, Faculty of Health and Medical Sciences, University of Copenhagen, Kopenhagen, Dänemark

**Keywords:** Vorhofflimmern, Herzrhythmusstörung, MHealth, Photoplethysmographie, Atrial fibrillation, Heart rhythm disorders, Mobile health, Photoplethysmography

## Abstract

Basierend auf der Technologie der Photoplethysmographie (PPG) lässt sich mit der Kamera eines Smartphones das Vorliegen von Herzrhythmusstörungen und die Herzfrequenz valide erfassen. Diese Technologie wurde im Rahmen des TeleCheck-AF-Projektes benutzt, um die effektive Durchführung von Telekonsultationen bei Patienten mit Vorhofflimmern zu ermöglichen. Die vorliegende Arbeit soll eine Übersicht über die PPG-Technologie und eine Schritt-für-Schritt-Anleitung für die Analyse und Interpretation der PPG-Signale bieten. Damit soll eine Integration und Implementierung dieser vielversprechenden und weit verfügbaren Technik in den klinischen Alltag gebahnt werden.

## Abstract (en)

By applying photoplethysmography (PPG), the camera of the mobile phone can be used to remotely assess heart rate and rhythm, which was widely used in conjunction with teleconsultations within the TeleCheck-AF project during the coronavirus disease 2019 (COVID-19) pandemic. Herein, we provide an educational, structured, stepwise practical guide on how to interpret PPG signals. A better understanding of PPG recordings is critical for the implementation of this widely available technology into clinical practice.

Die Therapie und Diagnostik von Herzrhythmusstörungen nehmen einen großen Stellenwert in der stationären und ambulanten Kardiologie ein. Insbesondere das Vorhofflimmern gehört dabei zu den Erkrankungen mit einer hohen Inzidenz, steigenden Prävalenz und zunehmender gesundheitsökonomischer Relevanz [1]. Gleichzeitig beginnt auch in Deutschland ein Wandel der Versorgungslandschaft durch einen Ausbau der Telemedizin [2]. Insbesondere im Bereich des Screenings auf Vorhofflimmern empfehlen aktuelle Konsensusdokumente den Einsatz sog. Smart Devices [3].

In Deutschland benutzen in etwa 77 % der Gesamtbevölkerung regelmäßig ein Smartphone, welches zur Beurteilung des Herzrhythmus in einer breiten Population benutzt werden kann [2, 4]. Verschiedene validierte Apps stehen aktuell als CE-markiertes, medizinisches Device zur Verfügung. Basierend auf der Photoplethysmographie(PPG)-Technologie, lässt sich mit der Kamera eines Smartphones beispielsweise das Vorliegen von Herzrhythmusstörungen valide erfassen [2, 5]. Aktuelle Daten zeigen eine hohe Sensitivität und Spezifität zur Detektion von Vorhofflimmern durch PPG-Analysen [5–9]. Die aktuellen Leitlinien der Europäischen Gesellschaft für Kardiologie (ESC) zur Diagnostik und Therapie von Vorhofflimmern schreiben für die Diagnose eines Vorhofflimmerns dessen Nachweis mittels 12-Kanal- (10 s), oder Ein-Kanal-EKG-Registrierungen (30 s) vor [10]. Daher eignet sich die PPG-Technologie insbesondere dazu, bei PatientInnen mit bereits diagnostizierten Herzrhythmusstörungen, wie z. B. Vorhofflimmern, telemedizinisch die Frequenz und den Rhythmus zu beurteilen, um die Behandlung entsprechend anzupassen [11]. Trotz der raschen Verbreitung von PPG-basierten Technologien fehlt bislang eine strukturierte Einweisung von ärztlichem und medizinischem Personal in deren Anwendung. Die damit verbundenen Unsicherheiten können dazu führen, dass die Technologie, obwohl sinnvoll, nicht eingesetzt wird [11].

*Telecheck-AF* ist ein internationales, multizentrisches Projekt zur Implementierung einer telemedizinischen Infrastruktur für die Versorgung von PatientInnen mit Vorhofflimmern durch Telekonsultation und „on-demand“ Herzfrequenz- und Rhythmusmonitoring mit Hilfe der validierten, CE-gekennzeichneten, PPG-basierten App Fibricheck® (Qopium, Hasselt, Belgien; [11–18]).

Die vorliegende Arbeit, basierend auf Erkenntnissen aus diesem Projekt [11, 13–18], soll eine Übersicht über die PPG-Technologie und eine Schritt-für-Schritt-Anleitung für die Analyse und Interpretation der PPG-Signale bieten. Damit soll eine Integration und Implementierung dieser vielversprechenden und weit verfügbaren Technik in den klinischen Alltag gebahnt werden [11].

## PPG-Technologie

**Figure 1:**
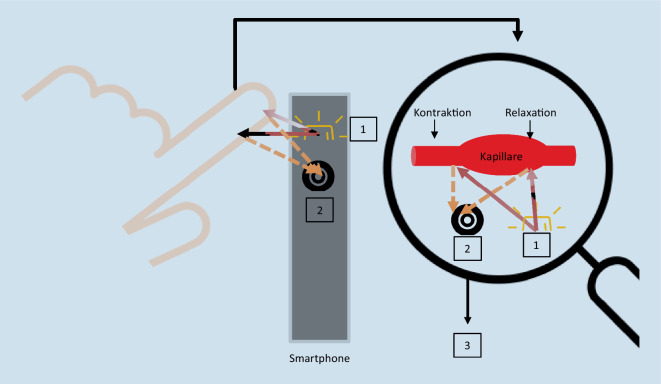

**Figure 2:**
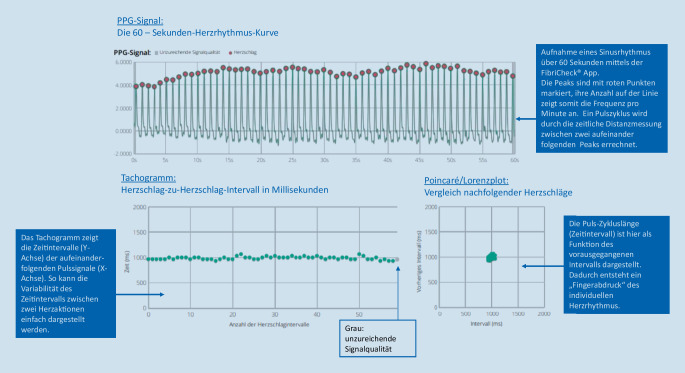

Für die PPG-Pulsmessung mittels Smartphone wird ein Finger vor das Blitzlicht und die Kamera gehalten, so dass das eindringende Licht an den Kapillaren pulsatil reflektiert und von der Kamera wieder erfasst werden kann (Abb. 1;[2, 12, 19]). Durch die Software wird das Videomaterial in eine Pulskurve transformiert [2, 11, 12, 20]. Die Zeitintervalle zwischen den einzelnen Peaks geben Auskunft über die Pulsregularität (Abb. 2; [12]). Zusätzlich erfolgt eine Darstellung des Rhythmus mittels Tachogramm und Poincaré/Lorenz-Plotdiagramm (Abb. 2), wodurch sich ein individueller Fingerabdruck des Herzrhythmus in einer übersichtlichen Form ergibt [11].

In den vorliegenden Beispielen wurde die Smartphone App Fibricheck® genutzt. Der Fibricheck®-Algorithmus (Fibricheck®, Qompium, Hasselt, Belgium) zeigt im Vergleich zu einem herkömmlichen 12-Kanal-EKG in Studien eine hohe Sensitivität und Spezifität zur Erkennung von Vorhofflimmern [12]. Auch im direkten Vergleich der Pulsanalyse von PatientInnen mit normalem Herzrhythmus und Vorhofflimmern zeigte sich eine hohe Korrelation [20]. Bei anderen Apps kann es zu leichten Unterschieden in Art der Darstellung der Daten kommen. Aufgrund der gleichen Funktionsweise im Bereich des PPG-gesteuerten Rhythmus-Monitorings via Smartphone ist die hier vorliegende Anleitung jedoch leicht übertragbar.

## PPG-Diagnostik Schritt für Schritt (Abb. 3)

**Figure 3:**
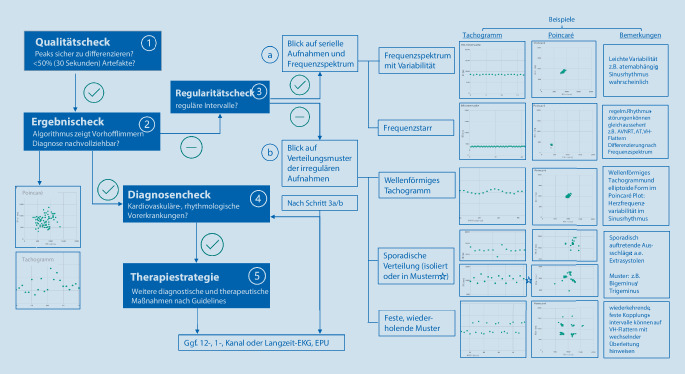

**Figure 4:**
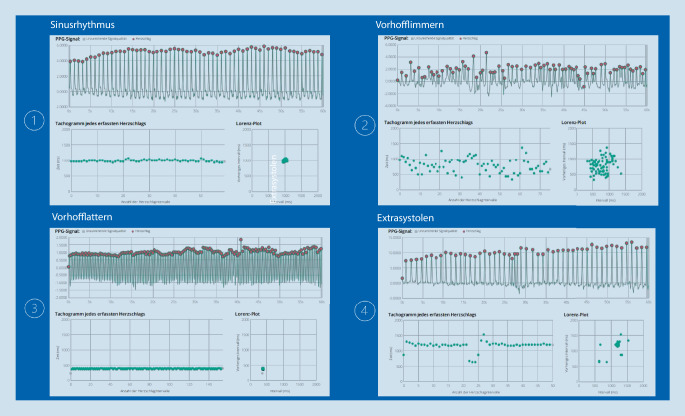

1. *Qualitätscheck:*
  Ein wichtiges Qualitätsmerkmal ist die gute Unterscheidbarkeit der unterschiedlichen Peaks. Zeigen sich verrauschte oder überlagerte Signale und Artefakte, kann eine Interpretation der Aufnahme erschwert sein. Wenn mehr als 50 % der Aufnahme (entsprechend 30 s) gestört ist, ist eine verlässliche Interpretation nicht möglich. Der Fibricheck®-Algorithmus erkennt jedoch Abschnitte mit niedriger Aufnahmequalität automatisch und markiert diese in grau. Peaks mit niedriger Aufnahmequalität werden blau dargestellt. Sobald sich Hinweise für eine minderwertige Aufnahme zeigen, werden die PatientInnen durch die App umgehend informiert, sodass eine Wiederholung stattfinden kann. Während des *TeleCheck-AF*-Projektes zeigten sich in nur 9,7 % der Fälle qualitativ niedrige Aufnahmen [11, 14, 17].
2. *Ergebnischeck:*
  Trotz der bereits nachgewiesenen hohen Sensitivität und Spezifität des Fibricheck®-Algorithmus zur Detektion von Vorhofflimmern [12] empfiehlt sich eine visuelle Kontrolle des Ergebnisses, da es bei jedem Algorithmus auch zu Fehldiagnosen kommen kann. Ein irregulärer Rhythmus, wie z. B. das Vorhofflimmern, zeigt sich üblicher Weise durch eine Irregularität der Intervalle zwischen den Peaks (Abb. 4). Dies resultiert in einer diffusen Verteilung der Punkte im Tachogramm und Poincaré/Lorenz Plot Diagramm. In Letzterem fehlt das bei einem regulären Rhythmus typische Clusterbild (Abb. 4 Sinusrhythmus/Vorhofflattern). Zeigen sich Zweifel an der durch Fibricheck angegebenen Diagnose, folgt Schritt 3 [11].
3. *Regularitätscheck:*
  Ein regelmäßiger Abstand der Peaks, eine geradlinige Darstellung auf dem Tachogramm sowie eine dichte Clusterbildung im Poincaré/Lorenz-Plotdiagramm zeigen einen regulären Rhythmus an (Abb. 4). Alle Abweichungen sind als Hinweise auf Irregularität zu werten und müssen gesondert beurteilt werden [11].
  - *Differenzierung eines regelmäßigen Rhythmus:* Bei einigen Herzrhythmusstörungen mit regelmäßigen RR-Intervallen, wie z. B. Vorhofflattern, regelmäßiger Vorhoftachykardie, AVNRT, AVRT und hämodynamisch stabiler VT, ist die rein PPG-basierte Interpretation und Unterscheidung zwischen einer regulären Rhythmusstörung und einem Sinusrhythmus erschwert [11, 12]. Speziell bei Rhythmusstörungen mit normofrequenten ventrikulären Schlägen (wie z. B. bei normofrequentem Vorhofflattern) kann in der Regel nicht sicher durch die PPG-Messung differenziert werden [11, 12]. Häufig kann die weitere Evaluation von mehreren aufeinanderfolgenden Messungen Informationen über den zugrunde liegenden Rhythmus genutzt werden.
    Ein Sinusrhythmus zeigt dabei in aufeinanderfolgenden Aufnahmen in der Regel keine identische Frequenz und auch eine Atemvariabilität (Abb. 4). Liegt in aufeinanderfolgenden Messungen aber anhaltend eine *identische Frequenz* vor, ist eine regelmäßige Herzrhythmusstörung zu vermuten (Abb. 4; [11]). Für die weitere Unterscheidung regelmäßiger Herzrhythmusstörungen eignet sich ein Blick auf das Frequenzspektrum: Bei einem Vorhofflattern liegt die typische Frequenz je nach Überleitung bei 120–160/min (2:1 Überleitung), 100/min (3:1 Überleitung) oder 75/min (4:1 Überleitung). Zudem ist die Angabe von Symptomen wie Palpitationen durch den Patienten in der Fibricheck®-App ein wichtiger Indikator für eine mögliche zugrunde liegende, regelmäßige Herzrhythmusstörung. In manchen Fällen ist letztendlich eine definitive Diagnose mittels PPG-Messung nicht immer möglich [11]. Bei unklaren Fällen sollte eine 12-Kanal- oder Langzeit-EKG-Untersuchung oder elektrophysiologische Untersuchung diskutiert werden. Hier können die PPG-basierten Aufnahmen und der Symptombericht der PatientInnen jedoch zur Indikationsstellung genutzt werden.
  - *Differenzierung eines unregelmäßigen Rhythmus:* Im ersten Schritt sollte darauf geachtet werden, ob trotz Irregularität dennoch ein Sinusrhythmus mit erhöhter Herzfrequenzvariabilität vorliegt. Eine erhöhte Herzfrequenzvariabilität zeigt eine Wellenform im Tachogramm und eine elliptoide Form im Poincaré/Lorenz-Plotdiagramm (Abb. 3). Besteht ein unregelmäßiger Rhythmus aufgrund intermittierender Extrasystolen, ist es wichtig, zwischen einem sporadischen und wiederkehrenden Muster zu unterscheiden. Zeigt sich ein unregelmäßiger Rhythmus nur sporadisch, können sich dahinter supraventrikuläre oder ventrikuläre Extrasystolen verbergen (Abb. 3 und 4). Diese können sich bei festen Kopplungsintervallen durch verkürzte Intervalle zwischen den Peaks (Extrasystole) mit nachfolgend kompensatorischer Intervallverlängerung (Pause) in den PPG-Rohdaten oder dem Tachogramm zeigen. Im Poincaré/Lorenz-Plotdiagramm zeigen sich bei Schlägen mit gleichem Kopplungsintervall besonders dichte Clustermuster [11]. Zeigen sich sporadisch auftretende Muster, kann es sich um eine Episode eines Bigeminus oder Trigeminus handeln. Bei tendenziell anhaltenden, unregelmäßigen Mustern sind mehrere Differenzialdiagnosen zu bedenken. Bei einem bradykarden Rhythmus ist an eine Bradyarrhythmie auf dem Boden von blockierten Extrasystolen durch variable Überleitung einer supraventrikulären Tachykardie zu denken. Zudem kann auch ein Vorhofflattern mit wechselnder Überleitung und festem Kopplungsintervall eine Musterbildung zeigen. Im PPG-Signal ergeben sich dann Peaks, die den vorausgehenden sehr rasch und zusammenhängend nachfolgen (Abb. 3). Das Tachogramm zeigt zwei oder drei getrennte Linien und im Poincaré/Lorenz-Plotdiagramm finden sich mehrere verschiedene, dichte Cluster. Auch in diesen Fällen benötigt man für eine sichere Differenzierung eine weiterführende Diagnostik, z. B. mittels Holter‑, EKG oder EPU [11].
4. *Was sind die kardiovaskulären und rhythmologischen Vordiagnosen?*
  Wie zuvor beschrieben, können durch PPG-basierte Diagnostik verschiedene Rhythmusstörungen nicht sicher differenziert werden. Neben den o. g. Beispielen, ist auch das Monitoring bei sehr tachykarden Rhythmusstörungen mit Vorsicht vorzunehmen, da aufgrund eines Pulsdefizites Fehldiagnosen entstehen können [11]. Neben der genannten Schritte (3a und 3b) zur weiteren Evaluation kann auch das Wissen über die kardiovaskulären und rhythmologischen Vorerkrankungen der Patienten helfen. In solchen Fällen ist es durchaus möglich, die PPG-Technologie auch auf das Monitoring von anderen Herzrhythmusstörungen, je nach Erfahrung und Vordiagnosen, auszuweiten [11].
5. *Fortsetzung der weiteren Diagnostik und Therapie*
  Zuletzt ist, je nach Indikation der anfänglichen Diagnostik, eine Fortsetzung der weiteren therapeutischen und diagnostischen Maßnahmen nötig [11, 21].

## Fazit und Perspektive

Die vorliegende Anleitung zur Evaluation und Interpretation von PPG-basierten Rhythmusanalysen soll den Einstieg in den Bereich des telemedizinischen Monitorings von Herzrhythmusstörungen via Smartphone (am Beispiel von Fibricheck®) erleichtern. Im Rahmen des multizentrischen, internationalen *Telecheck-AF*-Projekts erfolgte die Implementation einer telemedizinischen Infrastruktur zum PPG-basierten Monitoring von Patienten mit bekanntem Vorhofflimmern in verschiedenen klinischen Szenarien. Hier konnte gezeigt werden, dass Patienten motiviert sind, eine App für 7 Tage vor einer geplanten Telekonsultation „on-demand“ zu benutzen. Dabei war das Alter keine Limitation. Das mediane Alter war 65 Jahre und der älteste Patient war 92 Jahre alt [11, 14, 16, 17]. Das *Telecheck-AF*-Projekt ist auch an einigen deutschen Krankenhäusern erfolgreich implementiert, und es erfolgt zurzeit eine retrospektive Datensammlung und Analyse [11]. Auch wenn der klinische Nutzen sich bereits abzeichnet, steht eine flächendeckende Implementation einer solchen telemedizinischen Infrastruktur, wie in *TeleCheck-AF* beschrieben, in Deutschland noch vor einigen Herausforderungen.

Es bleibt spannend zu sehen, wie sich die telemedizinische und mHealth-basierte klinische Landschaft in naher Zukunft auch in Deutschland verändern wird. Weitere Studien zur Validierung und Implementierung von telemedizinischen Infrastrukturen sind hier noch nötig.
